# Schistosomal appendicitis in Qatar: a rare parasitic cause of acute appendicitis in a non-endemic region

**DOI:** 10.1093/jscr/rjaf1048

**Published:** 2026-01-08

**Authors:** Kamran Malik, Abdulbari Alsaeed, Hatim AlJumaili, Nabiha Malik, Ahmad Zarour

**Affiliations:** Department of Acute Care Surgery, Hamad General Hospital, Al Rayyan Road, Hamad Medical City, Al Rumailah, Doha, PO Box 3050, Qatar; Department of Medical Education, Hamad Medical Corporation, Al Rayyan Road, Hamad Medical City, Al Rumailah, Doha, PO Box 3050, Qatar; Department of Acute Care Surgery, Hamad General Hospital, Al Rayyan Road, Hamad Medical City, Al Rumailah, Doha, PO Box 3050, Qatar; Medical School, University of Limerick, Plassey, Castletroy, Co. Limerick, V94 T9PX, Ireland; Department of Acute Care Surgery, Hamad General Hospital, Al Rayyan Road, Hamad Medical City, Al Rumailah, Doha, PO Box 3050, Qatar

**Keywords:** schistosomal appendicitis, schistosomiasis, calcified ova, appendectomy, acute appendicitis

## Abstract

Acute appendicitis is a common surgical emergency, but its parasitic causes are often overlooked, particularly in non-endemic regions. Schistosomiasis, which affects over 250 million people globally, can rarely lead to schistosomal appendicitis (SA). A 32-year-old Filipino woman presented with acute right iliac fossa pain, vomiting, and loose stools. Clinical and imaging findings were consistent with appendicitis, and she underwent a laparoscopic appendectomy. Histopathological analysis revealed multiple calcified *Schistosoma* ova in the appendiceal wall, confirming SA. The patient recovered uneventfully and was referred to the Infectious Diseases department for further management. This case represents the third reported instance of SA in Qatar. SA is clinically and radiologically indistinguishable from typical appendicitis and is usually diagnosed retrospectively via histopathology. SA should be considered in the differential diagnosis of appendicitis in migrants from endemic regions. Routine histopathological examination of appendectomy specimens is crucial for accurate diagnosis and appropriate postoperative care.

## Introduction

Acute appendicitis (AA) is one of the most common surgical emergencies worldwide. According to the Global Burden of Disease Study, the incidence of AA increased by 38.8% between 1990 and 2019, reaching ~17.7 million cases globally, with an incidence rate of 228 per 100 000 population [1]. Despite this rising incidence, both mortality and death rates have declined significantly by 21.8% and 46.2%, respectively, reflecting advancements in surgical care and health system infrastructure.

While the etiology of appendicitis is often idiopathic or related to luminal obstruction, parasitic infections represent an underrecognized cause, particularly in non-endemic regions. Schistosomiasis is a parasitic disease caused by blood flukes of the genus *Schistosoma*, affecting over 250 million people across 78 countries, with an estimated annual mortality of 280 000–500 000 [2]. The disease is primarily endemic in sub-Saharan Africa, Southeast Asia, and parts of South America and the Middle East.

Schistosomal appendicitis (SA) is a rare manifestation of schistosomiasis, even in endemic regions. The exact pathophysiology remains uncertain. One proposed mechanism involves deposition of *Schistosoma* eggs within the appendiceal wall, leading to a localized immune response, granulomatous inflammation, fibrosis, and eventual luminal obstruction. Other hypotheses include ischemic injury due to egg embolization or chronic mucosal irritation, resulting in secondary bacterial infection [3, 4].

Clinically and radiologically, SA is indistinguishable from conventional appendicitis, and diagnosis is often retrospective, confirmed only through histopathological examination of the excised appendix.

## Case report

A 32-year-old previously healthy Filipino woman presented to the emergency department with a 1-day history of non-radiating right iliac fossa pain, accompanied by one episode of vomiting and three episodes of loose stools. She denied fever, nausea, anorexia, urinary symptoms, or similar prior episodes. Her menstrual history was unremarkable, with her last period occurring 10 days prior.

On examination, she was hemodynamically stable and afebrile. Abdominal examination revealed localized tenderness in the right iliac fossa with a positive Rovsing sign. There were no signs of peritonitis or palpable masses. Laboratory results showed leukocytosis (white blood cell (WBC) 17.2 × 10^9^/L, neutrophils 14.6 × 10^9^/L), hemoglobin 11.7 g/dL, and C-reactive protein (CRP) 37 mg/L; liver function and other tests were within normal limits.

**Figure 1:**
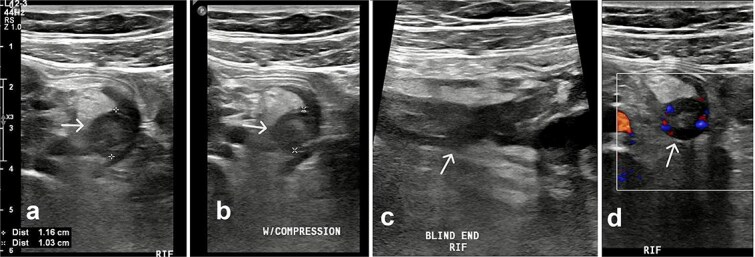
Ultrasound of the right iliac fossa. Transversal ultrasound image of the appendix (white arrow). (a) Before compression. (b) During compression, showing an uncompressible distended appendix (cursors). (c) At the level of the right iliac fossa, there is a blind-ending tubular structure.

Pelvic ultrasound demonstrated a non-compressible, blind-ended tubular structure in the right iliac fossa measuring 11 mm with increased wall vascularity, suggestive of acute appendicitis (Fig. 1).

**Figure 2:**
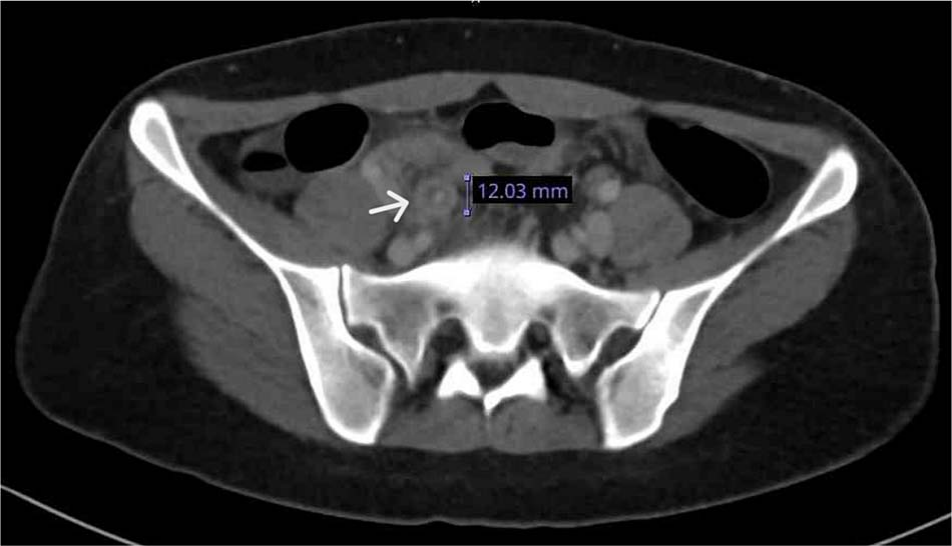
CT axial plane demonstrated a thickened appendix (white arrow) in the right iliac fossa measuring 12 mm.

A contrast-enhanced CT confirmed a thickened, inflamed appendix (12 mm diameter) with periappendiceal fat stranding and minimal pelvic fluid (Fig. 2).

**Figure 3:**
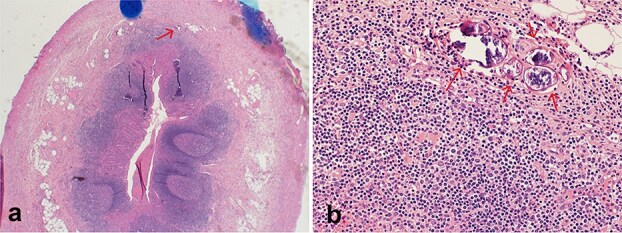
Histopathological section stained with H&E for the excised appendix: (a) (magnification 4×) an appendix shows acute appendicitis with transmural acute inflammation, a calcified Schistosoma egg is indicated by an arrow. (b) (magnification 40×) multiple calcified Schistosoma eggs in the submucosal layer of the appendix (arrows).

The patient underwent laparoscopic appendectomy. Intraoperatively, the appendix was inflamed with early adhesions to the anterior abdominal wall and a small volume of purulent pelvic fluid. Histopathological examination revealed transmural acute inflammation with multiple calcified *Schistosoma* ova in the mucosa and submucosa, confirming SA (Fig. 3).

The patient had an uneventful postoperative course and was discharged the next day with oral antibiotics and analgesia. At follow-up, she remained asymptomatic and was referred to the Infectious Diseases team for further management of schistosomiasis.

## Discussion

This case represents a rare instance of SA in a Filipino expatriate residing in Qatar, a non-endemic country. Despite the arid climate and absence of freshwater bodies that typically harbor *Schistosoma*-infected snails, Qatar has a high proportion of migrant workers (~88.4% of the population), many of whom originate from endemic regions such as the Philippines, Egypt, India, and Bangladesh. This demographic pattern increases the likelihood of imported parasitic diseases, including schistosomiasis.

Schistosomiasis is caused by five species of blood flukes, with *Schistosoma mansoni*, *Schistosoma japonicum*, and *Schistosoma haematobium* being the most clinically significant [2]. Although the disease predominantly affects the intestines and urogenital tract, appendiceal involvement is a recognized but rare complication. A 2021 systematic review reported a global prevalence of 1.31% for SA among appendectomy specimens, with higher rates in Africa (2.75%) compared to the Middle East (0.49%) [5].

In Qatar, this is the third reported case of SA, all involving expatriates from endemic countries (Table 1).

**Table 1:** Summary of reported cases of SA in Qatar.

Author	Year	Age	Sex	Ethnicity	Specimen parasite	Diagnosis	Outcome
Shankar Rao Buddhavarapu [6]	2008	28	F	Filipino	*S. japonicum*	Histological Examination	The patient left for the Philippines without undergoing further investigations.
Ali Toffaha [7]	2019	31	M	Tanzanian	Calcified ova	Histological Examination	Referred to the infectious disease clinic.
This case	2025	32	F	Filipino	Calcified ova	Histological Examination	Referred to the infectious disease clinic.

In each instance, diagnosis was made retrospectively through histopathological examination, underscoring the clinical indistinguishability of SA from standard acute appendicitis. In our case, species identification was not possible due to calcification of the ova, which is commonly observed in chronic infections.

Comparable regional data support this trend. A 5-year retrospective study in Kuwait (2007–2011) found eight cases of SA among 3012 appendectomy specimens, all involving Egyptian expatriates [8]. In Saudi Arabia, similar studies showed a decline in SA cases over time: 26 cases in 1987, 8 in 2012, and only 2 in 2015, despite comparable sample sizes [9–11]. While most cases involved Egyptian male laborers from endemic areas, reflecting the demographics of migrant workers in the region [12]. This suggests improvements in public health, reduced exposure to infested waters, and enhanced awareness.

The current case highlights broader public health challenges, including limited awareness and underdiagnosis of schistosomiasis in endemic regions like the Philippines, where sanitation infrastructure remains inadequate in rural areas [13]. Hasan *et al.* reported a 1.07% prevalence of SA in Egypt, emphasizing its rarity even in endemic settings [14].

Chronic schistosomal infection is associated with long-term complications, including bladder, hepatic, colorectal, and gastric malignancies. Persistent inflammation and immune-mediated tissue damage may contribute to carcinogenesis. Emerging evidence linking *S. japonicum* to colorectal cancer further supports early detection and management of SA, especially in young patients [15].

From a diagnostic standpoint, SA cannot be reliably differentiated from conventional appendicitis based on clinical presentation or imaging alone. Therefore, routine histopathological evaluation of appendectomy specimens is essential, particularly in migrant populations from endemic areas. Postoperative antiparasitic treatment with praziquantel remains the standard of care, even when diagnosis occurs outside endemic zones.

## Conclusion

SA is a rare cause of acute appendicitis and should be considered in patients from endemic regions, even in non-endemic countries like Qatar. Because clinical and imaging findings are indistinguishable from typical appendicitis, routine histopathological examination is essential for accurate diagnosis.

This case highlights the importance of maintaining awareness of imported parasitic infections in migrant populations. Early detection enables appropriate antiparasitic treatment and may reduce the risk of long-term complications. Strengthening diagnostic protocols and interdepartmental collaboration can improve outcomes in similar clinical settings.
